# Resumption of oral anticoagulation after spontaneous intracerebral hemorrhage

**DOI:** 10.1186/s42466-019-0018-0

**Published:** 2019-05-10

**Authors:** Jochen A. Sembill, Joji B. Kuramatsu, Stefan Schwab, Hagen B. Huttner

**Affiliations:** Department of Neurology, University Hospital Erlangen, Schwabachanlage 6, 91054 Erlangen, Germany

**Keywords:** Intracerebral hemorrhage, Oral anticoagulation, Resumption, Stroke prevention, Secondary prophylaxis, Intracranial hemorrhage

## Abstract

**Background:**

Given an ageing population the incidence of both patients suffering from intracerebral hemorrhage (ICH) and those requiring oral anticoagulation will increase. Up to now there are no results from randomized trials available whether or not, and when, ICH survivors should resume OAC. This review summarizes the most important observational studies, and initiated ongoing trials, to help guiding physicians in daily routine decision making.

**Findings:**

Several large observational studies and meta-analyses verified that OAC resumption was associated with a significant reduction of thromboembolic complications and mortality without leading to increased rates of recurrent ICH. OAC resumption seemed further associated with improved functional recovery and favorable long-term outcome. Given the general bleeding risk reduction in patients using Non–vitamin K antagonist oral anticoagulants (NOAC) compared to Vitamin-K-antagonist (VKA), NOAC use should also be preferred after ICH, although specific comparative studies are pending. Patients with lobar ICH need special attention as these patients showed increased ICH recurrence rates, why decision making should include extended diagnostic work-up evaluating cerebral microbleed burden, cortical subarachnoid hemorrhage and superficial siderosis. Further, patients with mechanical heart valves need specific consideration as restarting VKA may be unsafe until two weeks, whereas optimal balancing of hemorrhagic with thromboembolic complications may allow earlier re-initiation one week after ICH. In patients with atrial fibrillation, resumption generally should take place between 4 and 8 weeks after ICH depending on a patient’s individual risk profile. Left atrial appendage occlusion (LAAO) might represent an alternative strategy in high-risk patients. Ongoing clinical trials will clarify whether OAC resumption versus LAAO versus no antithrombotic therapy may represent the best possible secondary stroke prevention in ICH survivors with atrial fibrillation.

**Conclusions:**

According to observational data OAC resumption after ICH seems beneficial and safe. Ongoing clinical trials will create evidence regarding treatment effects of pharmaceutical resumption and interventional alternatives. Yet, individual decision making weighing the patient’s individual thromboembolic versus hemorrhagic risks remains essential.

## Background – intracerebral hemorrhage and oral anticoagulation

Intracerebral hemorrhage (ICH) still lacks effective treatments to positively influence functional outcome of patients suffering this severe sub-type of stroke (10–15%) [16, 39]. The consequences are high mortality rates (~ 50%) and functional dependency in many survivors (~ 2/3 of patients) [16, 39]. Although global incidence rates are expected to increase, the improved control of cerebrovascular risk factors may have contributed to a decline in the Western Hemisphere [23, 51]. As a potential future consequence hypertensive non-lobar ICH might occur less frequently than lobar ICH, the incidence of which is likely to rise with increasing rates of cerebral amyloid angiopathy (CAA) in an ageing society [5].

Likewise, rates of patients requiring oral anticoagulation (OAC) for prevention of thromboembolism due to atrial fibrillation (AF) are rising. Both, use of OAC and prior ICH, especially in lobar location, are known to increase the risk of recurrent intracerebral bleeding [6]. Both conditions occur coincidentally in almost one third of ICH patients - 15% of patients developed ICH already using OAC and another 15% have a de-novo diagnosis indicating future OAC prescription [24, 32, 34, 46]. Hence, question remains whether physicians should (re-)start a formally contraindicated treatment in high-risk patients already having experienced ICH as the most feared adverse OAC drug reaction.

### Anticoagulation resumption – a no-win situation?

The difficult decision whether or not to resume OAC after ICH is based on weighing patient’s individual risk for ischemic complications due to thromboembolism versus hemorrhagic complications, above all recurrent ICH. Due to low quality of evidence during time of preparation, the current 2014’s & 2015’s guidelines – American Heart Association/American Stroke Association, European Stroke Organization – do only provide little help, suggesting a multidisciplinary approach for individual decision making [16, 48]. While the benefit of OAC for prevention of thromboembolic complications, caused by several indications like atrial fibrillation, artificial heart valves, deep vein thrombosis and pulmonary embolism, or coagulopathies, is generally accepted, OAC resumption after ICH is mainly an issue of safety, i.e. risk of recurrent ICH [25].

Recurrence risk in general is related to several modifiable and non-modifiable factors. Patient age and stroke history represent non-modifiable risk factors for both thromboembolic and hemorrhagic complications, reflected by their simultaneous integration into commonly used stratification models (CHADS_2_-Score [0–6, from low to high stroke risk in AF patients; congestive heart failure =1, arterial hypertension =1, age ≥ 75 = 1, diabetes mellitus =1, previous stroke or transient ischemic attack =2] [12] and HAS-BLED-Score [0–9, from low to high major bleeding risk on anticoagulation; uncontrolled arterial hypertension =1, renal disease =1, liver disease =1, history of stroke =1, prior major bleeding =1, unstable INR =1, age > 65 = 1, use of drugs predisposing to bleeding =1, alcohol use =1]) [37]. The same holds true for (uncontrolled, > 140 mmHg) arterial hypertension representing a major – but importantly modifiable – risk factor especially for recurrence of ICH, increasing hazard ratios [HR] to 3.5 (95% CI(1.7–7.5), *p* = .001) in lobar ICH and to 4.2 (95% CI (1.0–17.5), *p* < .05) in non-lobar ICH [1]. Distinguishing location of index ICH is of outmost importance because of the strong relation between lobar ICH and cerebral amyloid angiopathy [25]. Lobar location increases the risk of recurrent ICH shown by longitudinal data (*n* = 1145) documenting a duplication of the annual recurrence rate compared to non-lobar ICH (7.8% versus 3.4%) [1]. In concordance, a meta-analysis of 9 cohort studies including 1552 patients investigated the first ever ICH risk in relation to cerebral microbleed status in patients with ischemic stroke and atrial fibrillation using long-term OAC [9]. The annual ICH incidence rate rose from 0.3% in patients without microbleeds to 0.8% in patients with any microbleeds and to 2.5% in patients with more than 5 microbleeds [9]. Moreover, a vast number of additional factors – gender, diabetes mellitus, serum lipid levels, smoking, alcohol or drug abuse, and further medication subtly interacting with coagulation and platelet function – have been documented to be associated with ICH recurrence, complicating the decision if, when and how to resume OAC after ICH [25].

### Current evidence

#### Resuming oral anticoagulation after ICH

After few and small-sized case series, the first larger retrospective observational analysis was published by Majeed et al. who investigated 234 patients with intracranial hemorrhage including 83 patients with ICH (55%, *n* = 83/234) documenting an overall increased risk for intracranial hemorrhage recurrence if OAC was resumed (11.5% versus 17.8%) [28]. In contrast, data from a Canadian registry (including 89% ICH patients, *n* = 252/284) reported annual recurrence rates less than 2.5% as well as a decreased mortality in patients resuming OAC [56]. Similar, a large Italian multicenter study documented an annual recurrence rate of 2.6% among 267 patients with intracranial hemorrhage resuming OAC (including 33% ICH patients, *n* = 88/267). All these studies remained inconclusive on whether or not risks for ICH recurrence were increased in a setting of OAC resumption [25].

From the year 2015 onwards there was growing evidence. The observational “geRman-widE mulTicenter Analysis of oRal Anticoagulation-associated intraCerebral hEmorrhage” (RETRACE) study included patients with OAC-associated ICH and investigated thromboembolic and hemorrhagic complication rates according to OAC exposure during one year of follow-up [24]. Among 719 survivors with AF, resumption of OAC significantly reduced thromboembolic events (OAC: 9/172 [5.2%] versus no-OAC: 82/547 [15.0%]; *p* < 0.001) without leading to increased rates of re-bleeding (OAC: 14/172 [8.1%] versus no-OAC: 36/547 [6.6%]; *p* = 0.48) [24]. Furthermore, OAC resumption was associated with a decreased long-term mortality risk among patients included in a propensity-matched survival analysis (HR: 0.258 (95% Confidence Interval [CI], 0.125–0.534; *p* < 0.001); i.e. 9 patients with OAC of 108 died (8.3%) compared to 47 patients without OAC of 153 (30.7%; *p* < 0.001) [24].

The same year, a large Danish registry including 1752 patients reported data strongly supporting these results [32]. The authors found a significantly decreased adjusted HR [0.55, 95% CI (0.39–0.78)] for all-cause mortality, stroke, and systemic embolism in patients on oral anticoagulant treatment in comparison with no treatment during 1-year follow-up [32]. The annual incidence rate of ischemic stroke and systemic embolism among patients using OAC was halved (5.3, 95% CI (3.3–8.5) per 100 patient years) compared with patients without antithrombotic treatment (10.4) or on antiplatelet therapy (10.3). For recurrent ICH, rates of 8.0 for OAC treated patients again did not significantly differ from 8.6 for patients with no antithrombotic treatment [adjusted HR, 0.91, 95% CI (0.56–1.49)], and 5.3 for patients using antiplatelet therapy [adjusted HR, 0.60, 95% CI (0.37–1.03)]. Another Danish population-based cohort study (*n* = 2978) confirmed these results, showing a significant lower risk of death [adjusted HR, 0.59, 95% CI (0.43–0.82)] and thromboembolic events [adjusted HR, 0.58 95% CI (0.35–0.97)] in ICH patients with post-discharge use of OAC, again without significantly increasing risks for major bleedings or recurrent ICH [adjusted HR 0.65, 95% CI (0.41–1.029] [34]. These results favoring resumption of OAC were further reproduced by several subsequent observational and registry studies [35, 36, 53].

To this day, the largest registry study was conducted in Taiwan and included 12,917 patients with intracranial hemorrhage from 1996 to 2011, reporting divergent results [4]. Chao and colleagues documented an increased risk for hemorrhage recurrence for both patients resuming OAC [HR 1.58, 95% CI (1.27–1.98)] as well as patients taking antiplatelet therapy [HR 1.36, 95% CI (1.19–1.57)] after propensity score matched analyses [4]. According to the authors only patients with a major thromboembolic risk (CHA_2_DS_2_-VASc-Score ≥ 6) would have a net-benefit resuming warfarin, shown by comparison of the number needed to treat of 27 (for preventing one ischemic stroke) versus the number needed to harm of 91 (for producing one ICH) [4]. However, these results might also be influenced by in general increased ICH risk in Asian patients [51].

Several meta-analyses of reported data have been conducted until now, all of them showing a significant reduction of thromboembolic complications without leading to increased risk of ICH recurrence [3, 22, 30, 57]. Furthermore, antiplatelet agents – sometimes considered as a safer alternative approach – were not beneficial neither for thromboembolism prophylaxis nor prevention of ICH recurrence [22]. One recent meta-analysis of individual patient data (*n* = 1012) did also address the association of OAC resumption with functional outcome, documenting that OAC resumption increases chances for a favorable outcome after 12 months (modified Rankin Scale 0–3) by 4-fold in both non-lobar and lobar ICH patients [2]. Even in the absence of recurrent clinically apparent stroke, patients resuming OAC seem to benefit with respect to better functional recovery, hypothetically due to prevention from micro- embolism cumulating to significant central nervous system damage influencing post-ICH recovery associated with cardioembolic stroke risk [29].

Of note, all of these observational studies harbor important limitations due to confounding by indication and selection bias [25]. Physicians individually weigh patient’s risk for ischemic versus hemorrhagic complications which results in selected patients with favorable risk-benefit-profiles restarting OAC. This might also be reflected by their younger age, less severe ICH, and better functional outcome in observational studies [24, 46]. In general, withholding therapy in severely affected patients is frequent in ICH care possibly further affecting post-discharge drug prescription [44, 46]. As statistical adjustment is to a large extent possible for quantifiable parameters, additional unmeasured variables likely introduce residual bias influencing the reported associations [22, 46]. Further, many investigations included heterogeneous patient cohorts combining different intracranial pathologies – mostly ICH, but also patients with subarachnoid hemorrhage, epidural or subdural hematomas – as well as OAC indications – atrial fibrillation, mechanical heart valves, deep vein thrombosis – each strongly influencing patients individual risk for recurrent hemorrhage or thromboembolism [25].

#### Mode of resumption

Although data from observational studies in the vast majority solely cover resumption of OAC using vitamin-K antagonists (VKA), it seems apparent that Non–vitamin K antagonist oral anticoagulants (NOAC) should be preferred [25]. Compared to VKA large randomized trials have demonstrated a halved ICH incidence in patients using NOAC [10, 20]. Although the mechanism behind risk reduction is not completely understood, it seems that the more selective mode of action, i.e. targeting only one clotting factor, together with limited crossing of the blood–brain barrier (dabigatran) or effluxing out of the brain by p-glycoprotein efflux pumps (rivaroxaban and apixaban) lead to a more beneficial safety profile [55]. Recently, a Bayesian network meta-analysis including 17 randomized controlled trials enrolling 116,618 patients evidenced that all NOACs were safer than warfarin for risk of ICH [55]. Relative ranking probability based on surface under the cumulative ranking curve (SUCRA) suggested the safest profile among NOACs for dabigatran (SUCRA, 0.86) followed by edoxaban (SUCRA, 0.81), apixaban (SUCRA, 0.61), and rivaroxaban (SUCRA, 0.32). VKA was ranked as the least safe drug among all anticoagulants (SUCRA, 0.06). In general, NOACs were associated with a significant 54% relative risk reduction compared with warfarin (Odds Ratio [OR] 0.46, 95% CI (0.35–0.59); *p* < 0.001). However, this analysis referred to drug safety only and did not focus on inter-class effects regarding prevention of thromboembolic events [55]. Available data for the comparison of severity of vitamin-K-antagonist related ICH with NOAC-ICH provide only little differences, however in-hospital mortality might potentially be reduced in ICH under NOAC [13, 19, 38, 49]. So far, studies specifically analyzing NOAC resumption after ICH do currently not exist [25].

#### Timing of resumption

The described observational studies documented a median starting point in between 4 to 6 weeks after ICH. Studies more specifically addressing this question reported a broad range of supposed optimal time points ranging from 72 h to 10–30 weeks [15, 28]. One large Swedish registry study (*n* = 2619) suggested an optimal time window within 7–8 weeks for resuming OAC after COX regression-based balancing between observed risk of ischemic and hemorrhagic complications [36]. Although having used sound statistical approaches, limitations of that study comprise censoring the first 4 weeks after ICH, narrow information on patient and ICH characteristics as well as treatment allocation gathered by outpatient dispensed drug registry [36]. A meta-analysis from the Kings College in London, UK, assessed the associations of resuming VKA six weeks after ICH with occurrence of both thromboembolic and hemorrhagic complications over a one-year follow-up time frame [22]. In essence, VKA-resumption was verified to be safe without increasing hemorrhagic complications over comparator treatments with platelet inhibitors [risk ratio: 1.34, 95% CI (0.79–2.30), *p* = 0.28], or no antithrombotic treatment respectively [risk ratio: 0.93, 95% CI (0.45–1.90), *p* = 0.84] [22]. As yet, it remains unclear when to optimally resume OAC after ICH but ongoing randomized trials might provide further evidence. Current expert opinion would suggest a timeframe between 4 to 8 weeks after index ICH depending on patient’s individual risk profile [25]. Application of a shorter time period to resumption may only be considered in life-threatening situations and compelling indications, such as symptomatic intracardiac thrombus formation or acute pulmonary embolism, and only after confirmation of hematoma stability by control imaging and strict blood pressure control.

#### Resumption in patients with mechanical heart valves

As mentioned above, NOAC should be preferred today for OAC resumption in indications like AF or venous thromboembolism. However, this does not apply for patients with mechanical heart valves (MHV) in situ as NOACs were shown to be inferior to VKA in these patients [11]. Neither cardiologic nor neurologic international guidelines provide specific recommendations how to treat MHV patients after ICH [16, 33, 48, 50]. Compared to AF, patients with MHV are at risk of increased thromboembolic complications why a recent consensus paper from the European Society of Cardiology Working Group in Thrombosis recommended that systemic anticoagulation using heparins may be safe to start as early as 3 days after ICH and oral anticoagulation using VKA after 7 days, based on limited data from small observational studies and case series [14].

**Fig. 1:**
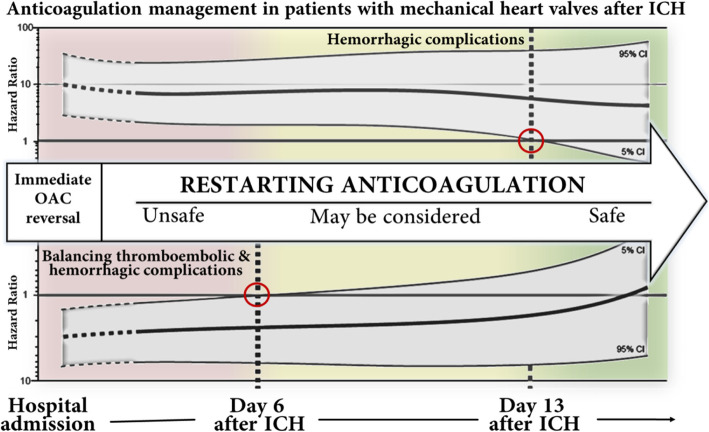
Suggested in-hospital anticoagulation management in patients with mechanical heart valves according to RETRACE analyses [26]. After initial reversal OAC should not be resumed before day 6 after ICH due to increased hazard for the composite of both thromboembolic and hemorrhagic complications. The hazard for hemorrhagic complications remained significantly increased until day 13. Suggested timeframes should be interpreted with respect to the patient’s individual thromboembolic and hemorrhagic risk. Figure modified after Kuramatsu et al., European Heart Journal 2018 [26]. Abbreviations: *CI* confidence interval, *ICH* intracerebral hemorrhage, *OAC* oral anticoagulation

The best available and most recent data came from a sub-group analysis of the German-wide multicenter RETRACE program which included among 2504 OAC-associated ICH patients 166 patients with MHV in situ [26]. Resumption of therapeutic anticoagulation, using heparins or VKA, was related to a 10-fold increase in incidence of major extra- or intracranial hemorrhagic complications during hospital stay in MHV-patients [rate ratio: 10.3, 95% CI (3.7–35.7)] [26]. Adjusted COX regression modeling for timing of anticoagulation revealed that OAC resumption may be safe after two weeks regarding bleeding complications, whereas optimal balancing of hemorrhagic with thromboembolic complications resulted in an earliest starting point of one week after ICH, to be considered especially in patients with highest risk for thromboembolism, i.e. concomitant AF, mitral position, or older prosthesis types [26]. Main results of this study are summarized in Fig. 1.

#### Resumption in patients with lobar ICH

Patients with ICH in lobar location compared to hypertensive non-lobar located ICH need special considerations due to strong relation to CAA, characterized by deposition of amyloid-β, micro-hemorrhages, and vascular fragility resulting in a greater risk for ICH recurrence [39, 45]. Genetically CAA shows association with apolipoprotein E alleles (subtypes epsilon 2 & 4) likely leading to amyloid deposition triggering recurrent ICH [16, 41]. A prediction model for CAA-associated lobar ICH integrating genetic characteristics – APOE ɛ4 carrier – and radiological CT parameters – subarachnoid hemorrhage, finger-like ICH projections – showed excellent discrimination [Area under the curve: 0.92, 95% CI (0.86–0.98)] in a recent prospective study [41]. Using MRI and clinical history CAA can be validly identified using the modified Boston criteria [27]. Observational studies documented recurrence rates for patients classified as having definite or probable sporadic CAA of 8.9 per 100 patient-years [95% CI (7.1–11)] [52].

MRI-detected presence and amount of cerebral microbleeds (CMB) seen on iron-sensitive sequences (T2*-weighted gradient-echo or susceptibility-weighted imaging) are associated with both first-ever ICH in ischemic stroke patients using OAC [OR 2.68, 95% CI (1.19–6.01), *p* = 0.017] as well as with recurrence of ICH [> 10 CMB versus none: OR 5.6, 95% CI (2.1–15), *p* = 0.001] [8, 9]. Pathologically, CMB correspond to hemosiderin deposits remaining in macrophages following a self-limiting microhemorrhage [47]. Therefore, they are seen as a neuroimaging marker for small vessel disease contributing to most lobar ICH [54]. Further, cortical superficial siderosis (cSS) and cortical or convexity subarachnoid hemorrhage (cSAH) were identified as independent marker for increased hemorrhagic risk [HR 3.92, 95% CI (1.38–11.17), *p* = 0.011, and HR 3.48, 95% CI (1.13–10.73), *p* = 0.030] [42]. If both probable CAA and cSS were present one investigation documented an ICH rate as high as 19% (95% CI, 11–32) compared to 6% (95% CI, 3–12) in patients without cSS during 5 years of follow-up [7].

**Fig. 2:**
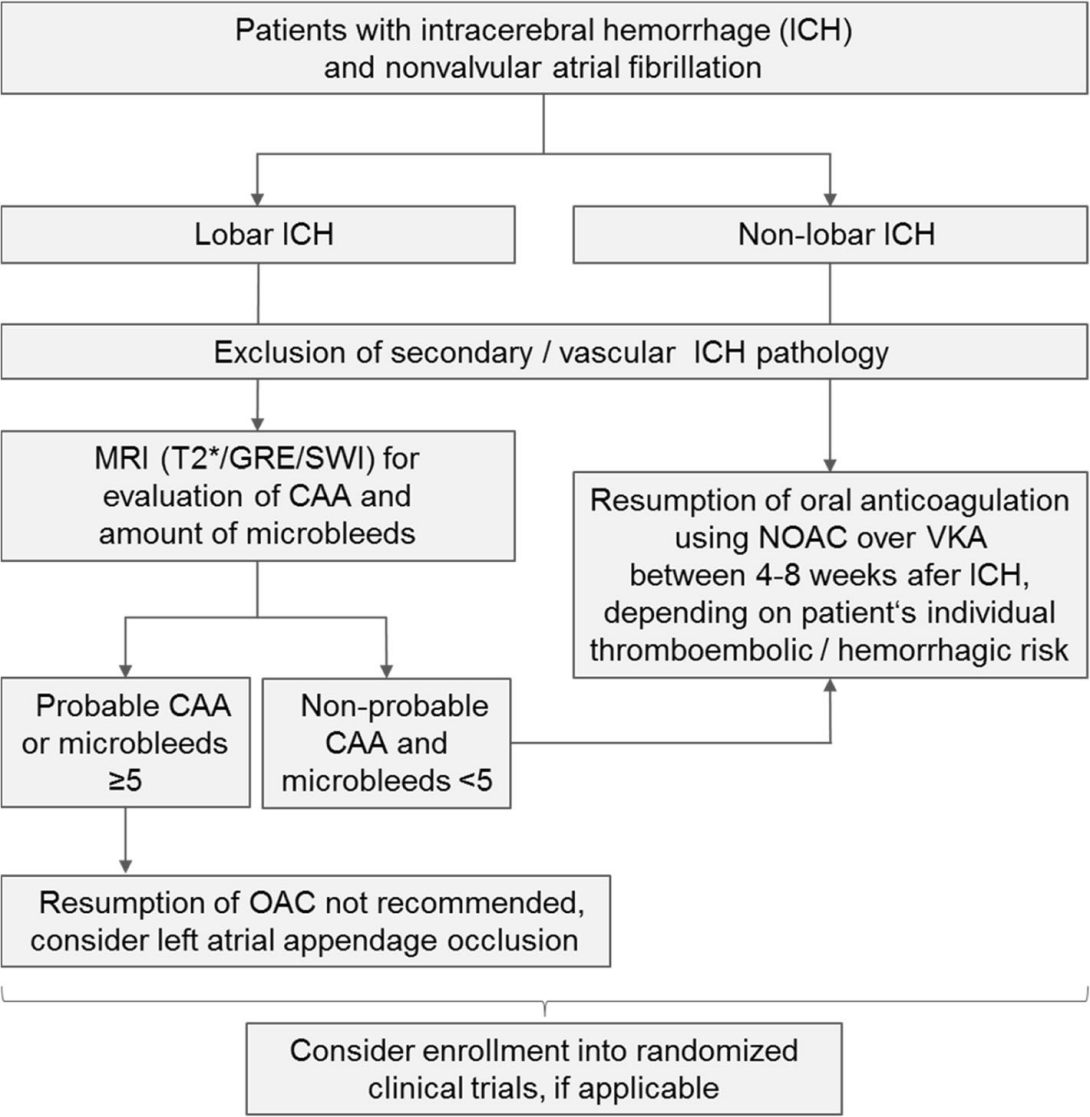
Suggested flow chart for anticoagulation resumption in ICH patients with nonvalvular atrial fibrillation. Abbreviations: *CAA* Cerebral amyloid angiopathy, *GRE* gradient echo, *MRI* Magnetic resonance imaging, *NOAC* Non–vitamin K antagonist oral anticoagulants, *OAC* Oral anticoagulation; Susceptibility-weighted imaging *SWI*, *VKA* Vitamin K antagonist

Only one sub-analysis of an individual patient data meta-analysis to date aimed to analyze the impact of OAC on ICH recurrence in patients with probable/possible CAA (*n* = 190). Although OAC resumption was consistent with overall lobar ICH associated with improved functional outcome and decreased mortality, numbers of patients and events were insufficient for investigating the influence of OAC on complication rates [2]. Summing up, decision making regarding OAC resumption in lobar ICH patients should include extended diagnostic work-up including magnetic resonance imaging to evaluate characteristics such as a microbleed burden, cSS or cSAH [25]. Their presence should lead to even more critical weighing of the potential benefit of OAC resumption versus the increased bleeding risk, considering also alternative interventional strategies for thromboembolism prevention [25]. For a suggested flow chart on OAC resumption in patients with deep compared to lobar ICH location please see Fig. 2.

#### Potential interventional prophylactic alternatives

The role of the left atrial appendage as the most important source of cardiac thromboembolism related to AF has led to introduction of left atrial appendage occlusion (LAAO) into clinical practice as a potential alternative treatment to long-term OAC. Percutaneous LAAO using the to date only approved device (WATCHMAN) showed non-inferiority compared to warfarin for prophylaxis of a primary composite endpoint of stroke, cardiovascular death, and systemic embolism in the multicenter, randomized PROTECT AF trial including 707 patients with non-valvular atrial fibrillation [18]. As several concerns were raised by the U.S. Food and Drug Administration (FDA) especially regarding early acute safety events, a second trial – PREVAIL – was performed, documenting an improved safety over time (7-day procedure-related complications: PREVAIL 4.2% versus PROTECT AF 8.7%, *p* = 0.004) [17, 18]. However, compared to warfarin this study did only reach non-inferiority regarding stroke or systemic embolism > 7 days [17]. Regarding the coprimary endpoint of the overall composite of stroke, systemic embolism and cardiovascular/unexplained death (18-month rate ratio 1.07 [0.57–1.89]) the upper bound of 1.89 extended the pre-specified noninferiority margin of 1.75, triggered by a higher number of (early) strokes in the intervention group (ischemic and hemorrhagic stroke, intervention: 6 [2.2%] versus control: 1 [0.7%]) [17]. Next to questionable efficacy and peri-interventional complications, the indicated antithrombotic therapy subsequent to device implantation needs consideration. The recommended antithrombotic therapy is related to the patient’s individual bleeding risk. Based on the PROTECT-AF trial protocol, patients of low bleeding risk should receive VKA for 45 days, and then switch to dual antiplatelet therapy until 6 months, followed by life-long low-dose aspirin monotherapy [18]. Patients of high bleeding risk, as among others patients after ICH, should be treated with dual antiplatelet therapy for one to six months, followed by life-long low-dose aspirin monotherapy. The assumed safety of this approach is based on results from the prospective multicenter nonrandomized ASAP feasibility study investigating LAAO in patients who were ineligible for OAC, however, safety in ICH patients remains unclear [40]. One observational study compared 151 ICH patients with AF who underwent LAAO with a propensity score-matched group of 151 patients receiving standard medical therapy [31]. Analyses showed a decreased risk for the composite endpoint consisting of all-cause mortality, ischemic stroke and major bleeding (HR 0.16, 95% CI (0.07–0.37)) as well as for recurrent ICH [HR 0.10, 95% CI (0.01–0.81)] in patients having LAAO [31]. Therefore, LAAO might potentially represent an alternative strategy to chronic OAC therapy in high-risk ICH patients, provided its successful evaluation in ongoing randomized-controlled trials – especially compared with NOAC as a safer and potentially more effective comparator than VKA [18, 21]. Today, according to FDA approval, interventional LAAO is formally contraindicated in patients with high-bleeding risk such as ICH patients and its off-label use should be preceded by a critical and interdisciplinary decision making process [43].

### Outlook

Several randomized controlled trials are currently registered in international trial registries investigating both pharmacological treatment and LAAO in patients after intracranial hemorrhage or with high bleeding risk, for overview see Table 1. Pharmacological treatment mostly consist of NOACs (APACHE-AF, ASPIRE, NASPAF-ICH, PRESTIGE-AF) compared to no antithrombotic drug or antiplatelets (all 1:1) whereas interventional trials are investigating LAAO versus NOAK (PRAGUE-17, 1:1; A_3_ICH, 1:1:1), or LAOO versus antiplatelets or none (ASAP-TOO, 1:1; A_3_ICH, 1:1:1), or LAAO versus best medical treatment (STROKECLOSE, 2:1; CLOSURE-AF, 1:1; LAAOS III, 1:1), or compare LAOO devices among each other (Amulet IDE, 1:1).

**Table 1:** Large randomized controlled trials investigating pharmacological or interventional treatment for stroke prevention after ICH

Trial name (ClinicalTrials.gov)	Design	Allocation ratio	Study population	Est. sample size (n)	Location	Intervention	Est. primary completion date
*Pharmacological treatment*							
APACHE-AF (NCT02565693)	Open label	1:1	ICH and AF	100	Netherlands	Apixaban vs antiplatelets or none	January 2021
ASPIRE (NCT03907046)	Quadruple-blind	1:1	Non-lobar ICH and AF	700	USA	Apixaban vs ASS 81 mg/d	April 2024
NASPAF-ICH (NCT02998905)	PROBE	1:1	ICH and AF	100	Canada	NOAC vs ASS 81 mg/d	October 2019
PRESTIGE-AF	Open label	1:1	ICH	662	Europe	NOAC vs antiplatelets or none	November 2022
SoSTART (NCT03153150)	PROBE	1:1	ICH and AF	800	United Kingdom	OAC vs antiplatelets or none	July 2021
STATICH (NCT03186729)	PROBE	1:1	ICH and AF or no AF	500	Scandinavia	OAC or antiplatelets vs none	June 2021
*Left atrial appendage occlusion (LAAO)*							
A_3_ICH (NCT03243175)	PROBE	1:1:1	ICH and AF	300	France	Apixaban vs LAAO vs antiplatelets or none	December 2022
Amulet IDE (NCT02879448)	Open label	1:1	High bleeding risk and AF	1878	Worldwide	Amulet LAAO vs WATCHMAN LAAO	February 2020
ASAP-TOO (NCT02928497)	Open label	2:1	High bleeding risk and AF	888	Belgium, Denmark, USA	LAAO vs antiplatelets or none	December 2023
CLOSURE-AF (NCT03463317)	Open label	1:1	High bleeding risk and AF	1512	Germany	LAAO vs active comparator (NOAK or VKA)	February 2021
LAAOS III (NCT01561651)	Quadruple-blind	1:1	Cardiopulmonary bypass surgery and AF	4812	Canada	Surgical LAAO vs best medical treatment	November 2022
PRAGUE-17 (NCT02426944)	Open label	1:1	History of bleeding and AF	400	Czech Republic	LAAO vs NOAK	May 2018
STROKECLOSE (NCT02830152)	PROBE	2:1	ICH and AF	750	Sweden	LAAO vs best medical treatment	May 2022

Information based on data from international (US, Asian, European) registries

Abbreviations: *AF* atrial fibrillation, *Est.* estimated, *ICH* inracranial hemorrhage, *LAAO* left atrial appendage occlusion, *PROBE* prospective randomized open blinded end-point

While some trials appear underpowered to detect significant differences (APACHE-AF, NASPAF-ICH, both *n* = 100), especially three well-designed large pharmacological trials may be able to document statistically significant effects of investigated treatments. First, the investigator-led “Start or STop Anticoagulants Randomised Trial” (SoSTART) plans to include 800 AF patients after non-traumatic and non-aneurysmal intracranial hemorrhage to compare treatment effects of any OAC versus antiplatelets or no antithrombotic medication on the incidence rate of a composite outcome of acute coronary syndrome, ischemic or hemorrhagic stroke, and vascular or non-vascular death. Second, the European Union funded “PREvention of STroke in Intracerebral haemorrhaGE survivors with Atrial Fibrillation” (PRESTIGE-AF) trial aims to include 662 patients with AF after ICH only to investigate superiority of NOACs for prevention of ischemic stroke and non-inferiority regarding recurrence of ICH compared to antiplatelets or no antithrombotic treatment. Third, the “Anticoagulation in ICH Survivors for PreventIon and REcovery” (ASPIRE) trial plans to include 700 ICH patients to analyze the hypothesis that Apixaban is superior to ASS for reducing rates of recurrent hemorrhagic or ischemic strokes or death. The strict exclusion of highest-risk patients – lobar ICH with cerebral amyloid angiopathy – prior identified by observational studies may represent a further advantage of this trial.

With respect to LAAO, two trials – STROKECLOSE and A_3_ICH – specifically include patients after ICH to compare LAAO with pharmacological treatments. Other trials will investigate patients with generally increased bleeding risk, i.e. high HAS-BLED-Scores, history of major bleeding, chronic kidney disease, or per investigator judgement. The German CLOSURE-AF study will analyze the incidence of a composite of stroke, systemic embolism, major bleeding, and cardiovascular or unexplained death, among 1512 patients with AF and high bleeding risk or history of bleeding (e.g. ICH), comparing LAAO with best medical care including NOAK or VKA. A different approach will be investigated by the quadruple-blind (participant, care provider, investigator, outcomes assessor) trial LAAOS III which will compare surgical LAAO with best medical treatment in patients with AF undergoing surgery for cardiopulmonary bypass.

Taken together, the near future will hopefully provide clear evidence created by large randomized trials to optimally treat patients with indication for oral anticoagulation after ICH.

## Conclusions

Sufficiently powered prospective randomized trials both investigating OAC resumption as well as LAAO are currently recruiting patients. To date, the best evidence comes from large observational studies and meta-analyses, indicating that resumption of OAC is reducing the risk for thromboembolic events and mortality without significantly increasing the risk for hemorrhagic complications. The use of NOACs might further contribute to safety of OAC resumption, which should take place between 4 to 8 weeks after ICH in patients with AF. Resumption of VKA in patients with MHV should not take place before one week after ICH. Safety may further be influenced by location of ICH and presence of CMB, cSS and cSAH, making it crucial to individually weigh patients’ risk for thromboembolic versus hemorrhagic complications.

## Abbreviations

AF: Atrial fibrillation
CAA: Cerebral amyloid angiopathy
CHADS: [Acronym] Congestive heart failure, Hypertension, Age, Diabetes mellitus, Stroke
CI: Confidence interval
CMB: Cerebral microbleeds
cSAH: cortical or convexity subarachnoid hemorrhage
cSS: cortical superficial siderosis
FDA: Food and Drug Administration
HAS-BLED: [Acronym] Hypertension, Abnormal renal/liver function, Stroke, Bleeding history or predisposition, Labile international normalized ratio, Elderly, Drugs/alcohol concomitantly
HR: Hazard ratio
ICH: Intracerebral hemorrhage
LAAO: Left atrial appendage occlusion
MHV: Mechanical heart valves
NOAC: Non–vitamin K antagonist oral anticoagulants
OAC: Oral anticoagulation
OR: Odds ratio
SUCRA: Surface under the cumulative ranking curve
VKA: Vitamin K antagonist
